# Activation of cannabinoid receptors in breast cancer cells improves osteoblast viability in cancer-bone interaction model while reducing breast cancer cell survival and migration

**DOI:** 10.1038/s41598-022-11116-9

**Published:** 2022-05-05

**Authors:** Tueanjai Khunluck, Kornkamon Lertsuwan, Chartinun Chutoe, Supagarn Sooksawanwit, Ingon Inson, Jarinthorn Teerapornpuntakit, Rutaiwan Tohtong, Narattaphol Charoenphandhu

**Affiliations:** 1 Center of Calcium and Bone Research (COCAB), Faculty of Science, Mahidol University, Bangkok, 10400, Thailand; 2 Faculty of Nursing, HRH Princess Chulabhorn College of Medical Science, Chulabhorn Royal Academy, Bangkok, Thailand; 3 Department of Biochemistry, Faculty of Science, Mahidol University, Rama VI Road, Bangkok, 10400 Thailand; 4 Department of Physiology, Faculty of Science, Mahidol University, Bangkok, Thailand; 5 Department of Physiology, Faculty of Medical Science, Naresuan University, Phitsanulok, Thailand; 6 Institute of Molecular Biosciences, Mahidol University, Nakhon Pathom, Thailand; 7 The Academy of Science, The Royal Society of Thailand, Bangkok, Thailand

**Keywords:** Cell migration, Apoptosis, Breast cancer

## Abstract

The endocannabinoid system has been postulated to help restrict cancer progression and maintain osteoblastic function during bone metastasis. Herein, the effects of cannabinoid receptor (CB) type 1 and 2 activation on breast cancer cell and osteoblast interaction were investigated by using ACEA and GW405833 as CB1 and CB2 agonists, respectively. Our results showed that breast cancer cell (MDA-MB-231)-derived conditioned media markedly decreased osteoblast-like UMR-106 cell viability. In contrast, media from MDA-MB-231 cells pre-treated with GW405833 improved UMR-106 cell viability. MDA-MB-231 cells were apparently more susceptible to both CB agonists than UMR-106 cells. Thereafter, we sought to answer the question as to how CB agonists reduced MDA-MB-231 cell virulence. Present data showed that co-activation of CB1 and CB2 exerted cytotoxic effects on MDA-MB-231 cells by increasing apoptotic cell death through suppression of the NF-κB signaling pathway in an ROS-independent mechanism. ACEA or GW405833 alone or in combination also inhibited MDA-MB-231 cell migration. Thus, it can be concluded that the endocannabinoid system is able to provide protection during breast cancer bone metastasis by interfering cancer and bone cell interaction as well as by the direct suppression of cancer cell growth and migration.

## Introduction

The endocannabinoid system (ECS) is a signaling network involved in several processes of human body, such as neurobehaviors, inflammation and bone metabolism^1–3^. Besides functioning physiological conditions, ECS has been postulated to be crucial for keeping the body within acceptable limits under certain pathological conditions, including bone metastasis^4,5^. Three fundamental components of the ECS are the endogenous cannabinoids (endocannabinoids), cannabinoid receptors (CB) and enzymes pertaining to endocannabinoid biosynthesis and degradation. The most common endocannabinoids namely, anandamide (AEA) and 2-arachidonoylglycerol (2-AG), exert their actions mainly through the activation of CB type 1 (CB1) and type 2 (CB2)^6^. Indeed, many cell types, e.g., embryonic stem cells, mesenchymal stem cells and osteoblasts, are found to express both CB1 and CB2^5,7–9^, and the final outcome from the ECS could be through the activation of both receptor types in an interdependent manner. In addition to its effects on the neural system, ECS plays an important role in bone homeostasis as well as interaction between cells in bone microenvironment^3,10–12^. Previous investigations on the dual roles of CB1 and CB2-mediated signaling in bone remodeling process have shown that the signaling pathways mediated through CB1 and CB2 were both needed to maintain stable bone mass.

Metastasis is a major cause of poor prognosis and death in cancer patients. Bone is a common metastatic site for several types of cancer, particularly prostate and breast cancers. Previous studies demonstrated that breast cancer induced osteoblast apoptosis and inhibited osteoblast cell adhesion and differentiation^13,14^. Hence, breast cancer can cause osteolytic bone lesion resulting in bone loss and cancer-induced bone pain. Meanwhile, ECS was also shown to be associated with cancer progression and metastasis. Both CB1 and CB2 expression was detected in many types of cancers, including breast cancer. Cancer development, cancer metastasis and tumor growth at both primary and metastatic sites were also correlated with the increased level of 2-AG and alteration of CB expression in tumor environment as well as in the plasma; thus, indicating the importance of ECS modulation in cancer growth and metastasis^15^. It is hypothesized that the elevated levels of endocannabinoids could play a role in immune regulation during cancer progression. Regardless, the expression of CB was shown to correlate with poor prognosis in breast cancer, and both CB1 and CB2 agonists, individually, were shown to suppress cancer growth both in vitro and in vivo^16,17^.

Both CB1 and CB2 are Gi protein-coupled receptors (GiPCRs). Signaling mediated from this group of receptors is known to inhibit adenyl cyclase activity leading to a reduction in cAMP production. For breast cancer, several studies in MDA-MB-231 and other breast cancer cell lines showed that the activation of CB1 or CB2 resulted in extracellular signal-regulated kinase (ERK)1/2 inhibition. Moreover, CB2 activation in breast cancer also led to protein kinase B (AKT)/mammalian target of rapamycin (mTOR) inhibition. Both ERK1/2 and AKT/mTOR pathways could lead to cancer cell growth suppression, ER stress and apoptosis as reviewed in Almeida et al., Mangal et al., and Caffarel et al.^18–20^. Among other downstream signaling molecules regulated by the two pathways, nuclear factor kappa B (NF-κB) was investigated further in this study due to its involvement in cancer cell progression as well as breast cancer and bone interaction^21–23^. Previous studies also showed that cannabidiol (CBD) increased reactive oxygen species (ROS) production in breast cancer cells via transient receptor potential cation channel subfamily V member 1 (TRPV1)^18,19^. However, whether the activation of CB1 and CB2 on breast cancer cells also resulted in increased ROS production was not known. Moreover, the production of ROS was reported to be associated with cancer progression and apoptosis induction (as reviewed in Aggarwal et al.)^24^. Elevated level of ROS in bone microenvironment was also associated with bone diseases in which it stimulated osteoclast differentiation while suppressing osteoblast differentiation^25^. Therefore, the potential involvement of cellular ROS production in CB-mediated breast cancer cell suppression was also investigated in this study.

While ECS was shown to regulate both cancer cell progression and bone homeostasis, it was not known whether this system also affected the interaction between cancer and bone cells during breast cancer bone metastasis. Since both CB1 and CB2 agonists were readily produced in bone environment as mentioned earlier, it was not known how cancer cells would respond to the concurrent presence of the two CB agonists. Accordingly, the present study aimed to investigate the potential effects of CB agonists on the interaction between breast cancer and osteoblast cells—i.e., a condition that mimicked metastasizing neoplastic cell-osteoblast interaction inside bone microenvironment. Since a positive outcome was observed in cancer-osteoblast interaction study, we also sought to explain the underlying mechanisms by which CB agonists suppressed the virulence of breast cancer cells. In this study, synthetic CB agonists were used to represent different types of CB in bone microenvironment, and MDA-MB-231 and UMR-106 cells were used to represent breast cancer and bone-forming cells (osteoblast), respectively.

## Materials and methods

### Cell culture

Osteoblast-like UMR-106 cells (RRID CVCL_3617; ATCC CRL-1661) and triple-negative breast cancer cells, MDA-MB-231 (RRID CVCL_0062; ATCC HTB-26) were obtained from American Type Culture Collection (ATCC, VA, USA). Cells were cultured in Dulbecco’s modified Eagle’s medium (DMEM) (Sigma-Aldrich, MO, USA) containing 10% (v/v) fetal bovine serum (FBS) and 1% penicillin–streptomycin (Gibco, Texas, USA) and maintained under 5% CO_2_ at 37 °C, and sub-cultured according to the manufacturer’s instruction.

### Chemicals

CB1 and CB2 agonists—ACEA (A9719) and GW405833 (G1421), respectively—were purchased from Sigma-Aldrich. Antibodies against phosphorylated NF-κB p65 (p-NF-κB p65) (RRID AB_331284; #3033), caspase-3 (RRID AB_2069872; #9665), rabbit IgG-HRP (RRID AB_2099233; #7074) conjugated and mouse IgG-HRP conjugated (RRID AB_330924; #7076) were purchased from Cell Signaling Technology Inc (MA, USA). Primary β-actin antibody (RRID AB_476693; #A2066) was purchased from Sigma-Aldrich. All antibodies have been validated to show appropriate specificity and sensitivity.

### Preparation of conditioned media (CM)

**Figure 1:**
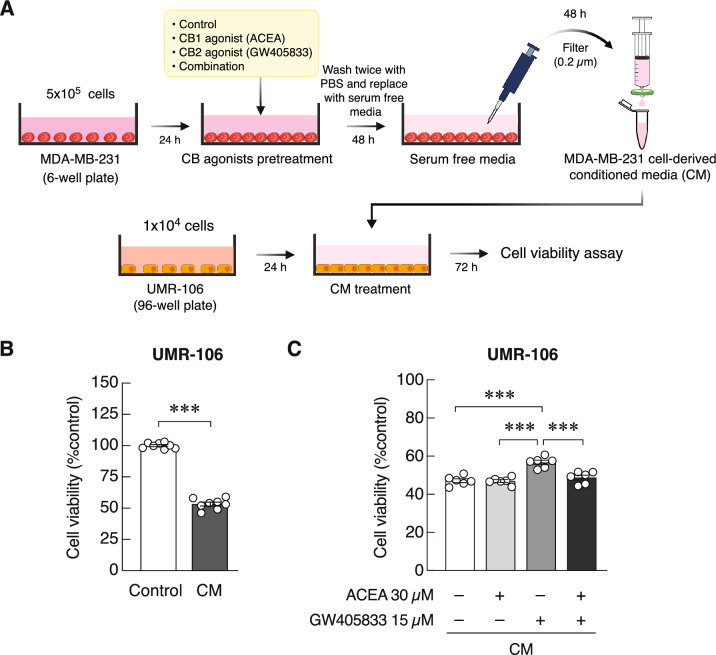
Effects of MDA-MB-231-derived CM and CM collected from MDA-MB-231 cells pretreated with ACEA, GW405833 or the combination of both on UMR-106 cell viability were illustrated. (**a**) Diagram illustrated experimental outline of this experiment. (**b**) Results of UMR-106 cell viability were represented as mean ± SEM from three independent biological replicates with two technical replicates each. (***p < 0.001).

To collect CM from MDA-MB-231 cells, cells were seeded into a 6-well plate at a density of 5.0 × 10^5^ cells/well and grown for 24 h until reaching 80–90% confluence. In the pretreatment experiment, cells were treated with either ACEA, GW405833 or a combination of both for 48 h. After that, cells were washed with phosphate buffered saline (PBS) and incubated in serum-free DMEM. Forty-eight hours later, CM from these cells was harvested, filtered through a syringe filter (pore size of 0.2 µm; Sartorius Stedim Biotech GmbH, Göttingen, Germany) to remove cells and debris, and stored at − 80 °C for later experiments (Fig. 1a).

### Cell viability assay

Cell viability was assessed using the 3-(4,5-dimethylthiazol-2-yl)-2,5-diphenyltetrazolium bromide (MTT) colorimetric assay. The assay was performed according to a previously described method^26^. In brief, cells were seeded into a 96-well plate at a density of 1.0 × 10^4^ cells/well and allowed to attach overnight. Thereafter, they were treated with ACEA, GW405833 or the combination of both for the indicated times. MTT (Invitrogen, CA, USA) solution was added to the final concentration of 0.5 mg/mL in 100 µL in serum-free DMEM and incubated at 37 °C for 4 h, then stop solution was added and thoroughly mixed. The optical density at 595 nm was measured using a microplate reader (Multiskan EX) (Thermo Fisher Scientific, MA, USA). The percentage of cell viability was calculated based on the absorbance ratio between cells treated with the compounds and control multiplied by 100 (percent cell viability normalized to control).

### Apoptosis assay

MDA-MB-231 cells were seeded into a 6-well plate at a density of 3.3 × 10^5^ cells/well and allowed to attach overnight. Thereafter, the cells were treated with ACEA, GW405833 or the combination of both for 48 h. At the end of the experiment, cells were stained with Annexin-V-FITC (A13199) (Invitrogen) and propidium iodide (PI) (P3566) (Invitrogen) according to the manufacturer’s guideline. Apoptotic cells were quantified using FACScan flow cytometer (BD FACSCanto) (BD Biosciences, CA, USA). Cells were analyzed for the percentage of cells in healthy, early apoptotic, late apoptotic and necrotic phases by using FACSDiva™ Software version 6.1.3 (BD Biosciences).

### DCFDA cellular ROS detection assay

Cellular reactive oxygen species (ROS) were monitored using the 2´,7´-dichlorofluorescin diacetate (DCFDA) Cellular ROS Detection Assay Kit (ab113851) (Abcam, Cambridge, UK) according to the manufacturer's instruction. In brief, MDA-MB-231 cells were seeded into a 96-black well plates with clear bottom at a density of 2.5 × 10^4^ cells/well and grown overnight. Then, the cells were treated with ACEA, GW405833 or the combination of both for 6 h. At the end of the treatment, fluorescence signal of DCF was measured by using a multimode microplate reader (Spark 10 M) (Tecan Austria GmbH, Männedorf, Austria) with the excitation and emission wavelengths of 485 and 535 nm, respectively.

### Western blot analysis

Twenty micrograms of protein samples were resolved by sodium dodecyl sulfate polyacrylamide gel electrophoresis (SDS-PAGE) (Invitrogen) gel before being transferred to nitrocellulose blotting membrane (GE Healthcare, Texas, USA).The membranes were then blocked with 5% (w/v) bovine serum albumin (BSA) (Capricorn Scientific, Hessen, Germany) for 2 h at room temperature, followed by the incubation at 4 °C overnight with primary antibodies. After washing, the membranes were incubated with the appropriate horseradish peroxidase (HRP)-conjugated secondary antibodies. Proteins on the immunoblots were visualized by enhanced chemiluminescence (ECL) (Millipore, MA, USA) and exposed to X-ray film (GE Healthcare). Protein band intensity was quantified using ImageJ software (National Institutes of Health, MD, USA). To relatively quantify the target protein expression, the intensity of target protein band was normalized to β-actin of each lane.

### Cell migration assay

Cell migration was measured by wound healing assay modified from Lertsuwan et al.,^27^. Briefly, MDA-MB-231 cells were seeded into a 24-well plate at a density of 4.0 × 10^5^ cells/well and allowed to attach overnight. Thereafter, wound was created with a sterile 200-µL pipette tip, then the cells were treated with ACEA, GW405833 or the combination of both. Series of images of the scratched wound were taken under a phase contrast inverted microscope (model AE31) (Motic, China) from 0 to 36 h. The closure of the wound representing migration ability of the cells was measured by using ImageJ software (National Institutes of Health). Cell migration was calculated as the percentage of wound closure area compared to the initial scratched area.

### Statistical analysis

All results are expressed as means ± standard errors (SEM). Unless otherwise specified, statistical analysis for multiple comparisons was performed using one-way analysis of variance (ANOVA). The difference between pairs of means was analyzed by Tukey post-test. A *p*-value < 0.05 was considered statistically significant. Calculation of the inhibitory concentration at 50% (IC_50_) and all statistical analyses were performed using GraphPad Prism 9 (GraphPad Software Inc., USA).

## Results

### MDA-MB-231 cell-derived CM inhibited osteoblast-like UMR-106 cell survival, while pretreatment of GW405833 prevented this effect

Since breast cancer cells appear to have a great propensity for bone metastasis, the ability of breast cancer cells to alter osteoblast viability was determined by indirect-contact experiment. We evaluated the indirect effect of MDA-MB-231 cell-derived CM on osteoblast-like UMR-106 cell survival using MTT assay (Fig. 1a). As shown in Fig. 1b, UMR-106 cell viability was significantly inhibited by 46.75% when cells were treated with fifty percent of MDA-MB-231 cell-derived CM as compared to those treated with fifty percent serum free media. To further elaborate the potential effects of CB1 and CB2 agonists on this interaction, CM collected from MDA-MB-231 cell-pretreated with ACEA, GW405833 or their combination were used. While pretreatment with ACEA or the combination did not alter CM-mediated osteoblast suppression, significant recovery was observed in UMR-106 cells treated with CM from MDA-MB-231 pretreated with CB2 agonist, GW405833 (Fig. 1c). It was thus suggested that the ECS—particularly neoplastic CB2 activation—was able to alleviate the negative effect of breast cancer cells on osteoblasts.

### High concentration of ACEA and GW405833 differentially suppressed UMR-106 cell and MDA-MB-231 cell viability

**Figure 2:**
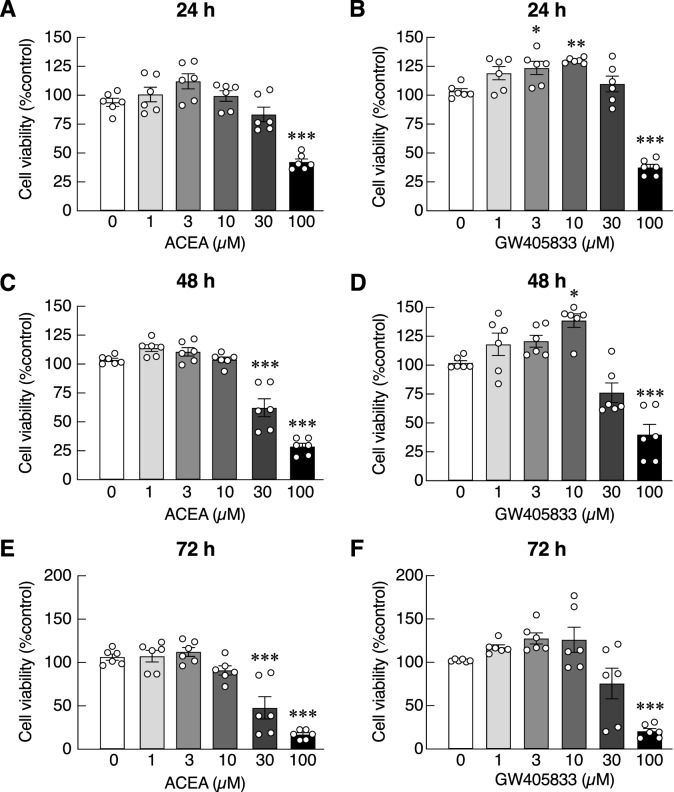
Cytotoxic effects of ACEA (**a**–**c**) and GW405833 (**d**–**f**) on UMR-106 cell viability were measured by MTT assay. Cells were treated with the compounds of various concentration (0–100 µM) for 24, 48 or 72 h. The results were expressed as mean ± SEM of three independent biological replicates with two technical replicates each. (***p < 0.001, **p < 0.01, *p < 0.05).

**Figure 3:**
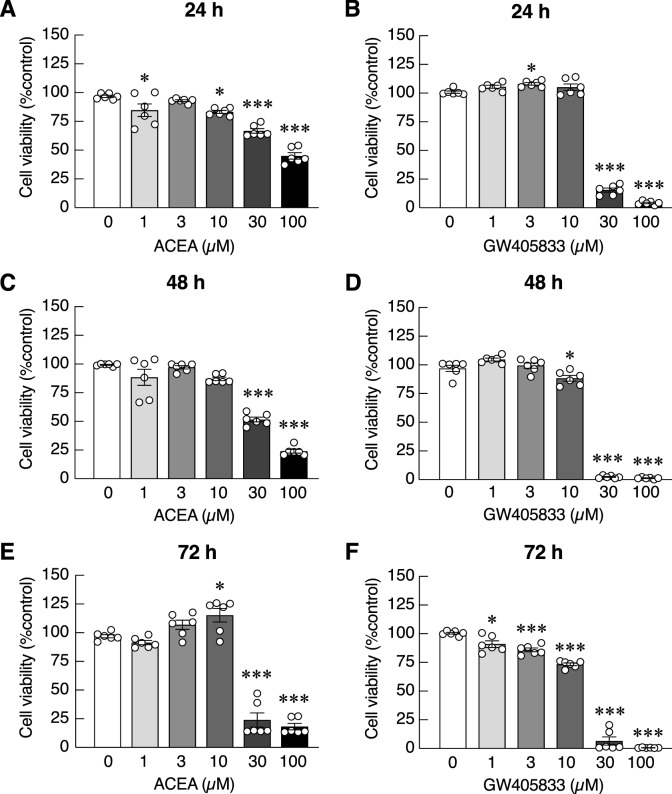
Cytotoxic effects of ACEA (**a**, **c**, **e**) and GW405833 (**b**, **d**, **f**) on MDA-MB-231 cell viability were measured by MTT assay. Cells were treated with the compounds of various concentrations (0–100 µM) for 24, 48 or 72 h. The results were expressed as mean ± SEM of three independent biological replicates with two technical replicates each. (***p < 0.001, *p < 0.05).

To examine direct effects of ACEA and GW405833 on UMR-106 and MDA-MB-231 cell viability, cells were treated with varying doses of CB agonists for 24, 48 and 72 h, before MTT assay. Dose-dependent inhibitory effects of ACEA and GW405833 on UMR-106 and MDA-MB-231 cell survival were observed with different IC_50_ (Figs. 2 and 3 and Tables 1 and 2). Specifically, UMR-106 exhibited markedly higher IC_50_ values reflecting its lower sensitivity to both ACEA and GW405833 than MDA-MB-231 (Table S1). For example, the IC_50_ values for ACEA at 48 h were 61.84 µM and 38 µM for UMR-106 and MDA-MB-231, respectively. Similar results were observed for GW405833 where the IC_50_ values for GW405833 at 48 h were 119.20 µM and 16.60 µM for UMR-106 and MDA-MB-231, respectively. Considering the sensitivity of these compounds against each type of cells, MDA-MB-231 cells were more sensitive to CB2 agonist (GW405833) than CB1 agonist (ACEA), while UMR-106 cells were more sensitive to CB1 agonist (Tables 1 and 2). On average, MDA-MB-231 cells were more sensitive to ACEA and GW405833 than UMR-106 cells by 1.44-fold and 6.76-fold, respectively (Table S1).

**Table 1:** The estimated IC_50_ values of ACEA and GW405833 on UMR-106 cell viability.

Compounds	IC_50_ value (µM)		
	24 h	48 h	72 h
ACEA	104.50	61.98	37.96
GW405833	168.10	119.20	79.86

The IC_50_ values were calculated using a nonlinear regression model from results in Fig. 2 by GraphPad Prism 9.1.2.

**Table 2:** The estimated IC_50_ values of ACEA and GW405833 on MDA-MB-231 cell viability.

Compounds	IC_50_ value (µM)		
	24 h	48 h	72 h
ACEA	67.81	38.00	32.83
GW405833	24.63	16.60	12.72

The IC_50_ values were computed using a nonlinear regression model from results in Fig. 3 by GraphPad Prism 9.1.2.

### Combination of ACEA and GW405833 augmented their cytotoxicity in MDA-MB-231 cells

**Figure 4:**
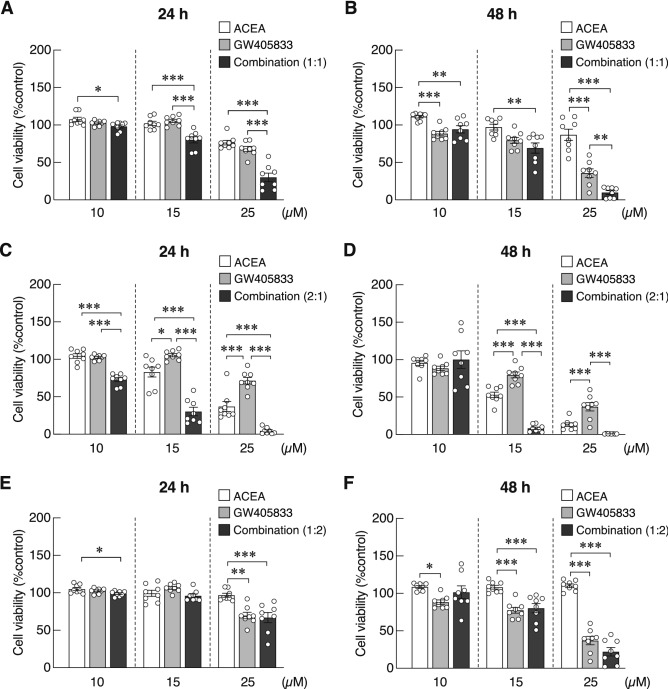
Combining effects of ACEA and GW405833 at different ratio on MDA-MB-231 cell survival were evaluated. Cell viability was determined by MTT assay after treatment with the combination of these compounds at different ratio (ACEA:GW405833) of 1:1 (**a**, **b**), 2:1 (**c**, **d**) or 1:2 (**e**, **f**) for 24 and 48 h. Bars represent mean ± SEM of four independent biological replicates with two technical replicates each. (***p < 0.001, **p < 0.01, *p < 0.05).

As seen in Fig. 3, exposure to specific CB1 or CB2 agonist alone significantly inhibited MDA-MB-231 cell viability. We therefore evaluated whether the co-activation of CB1 and CB2 receptors by ACEA and GW405833 at different combination ratio could enhance their cytotoxic effects. As GW405833 was apparently more toxic to MDA-MB-231 than ACEA (Fig. 3 and Table 2), the concentration close to the IC_50_ of GW405833 at each time point was used for the combination treatments. Hence, 10 µM, 15 µM and 25 µM GW405833, representing the IC_50_ at 72, 48 and 24 h, were used in the combination treatment. At ratio of 1:1 (ACEA:GW405833), 10 µM ACEA + 10 µM GW405833, 15 µM ACEA + 15 µM GW405833 and 25 µM ACEA + 25 µM GW405833 were used. At ratio of 2:1 (ACEA:GW405833), 20 µM ACEA + 10 µM GW405833, 30 µM ACEA + 15 µM GW405833 and 50 µM ACEA + 25 µM GW405833 were used. Lastly, at the ratio of 1:2 (ACEA:GW405833), 5 µM ACEA + 10 µM GW405833, 7.5 µM ACEA + 15 µM GW405833 and 12.5 µM ACEA + 25 µM GW405833 were used. Whereas a significant reduction was found when both 1:1 and 2:1 combination were applied as compared to the individual treatments, the enhancement was not seen at a combination ratio of 1:2 (Fig. 4). At the combination ratio of 1:1, a significant reduction of cell viability could be seen when GW405833 at 15 µM and 25 µM were used at 24 h as compared to individual treatments. This phenomenon was observed at the highest concentration at 48 h (Fig. 4a,b). More drastic enhancement was seen at the 2:1 combination. At 24 h, the combination treatments notably decreased MDA-MB-231 cell viability by 27.13%, 69.73% and 95.61% when GW405833 at 10 µM, 15 µM and 25 µM were used (Fig. 4c). Greater reduction in cell viability was shown when treatments with the combination of 30 µM ACEA + 15 µM GW405833 and 50 µM ACEA + 25 µM GW405833 were given at 48 h, i.e., 91.76% and 99.19%, respectively (Fig. 4d). Thus, the aforementioned results suggested that a concurrent exposure to CB1 and CB2 agonists—especially at the ratio of 2:1—considerably enhanced the cytotoxic effects on breast cancer MDA-MB-231 cells. This combination ratio was, therefore, selected for further experiments.

### Combination of ACEA and GW405833 enhanced MDA-MB-231 cytotoxicity by inducing apoptosis

**Figure 5:**
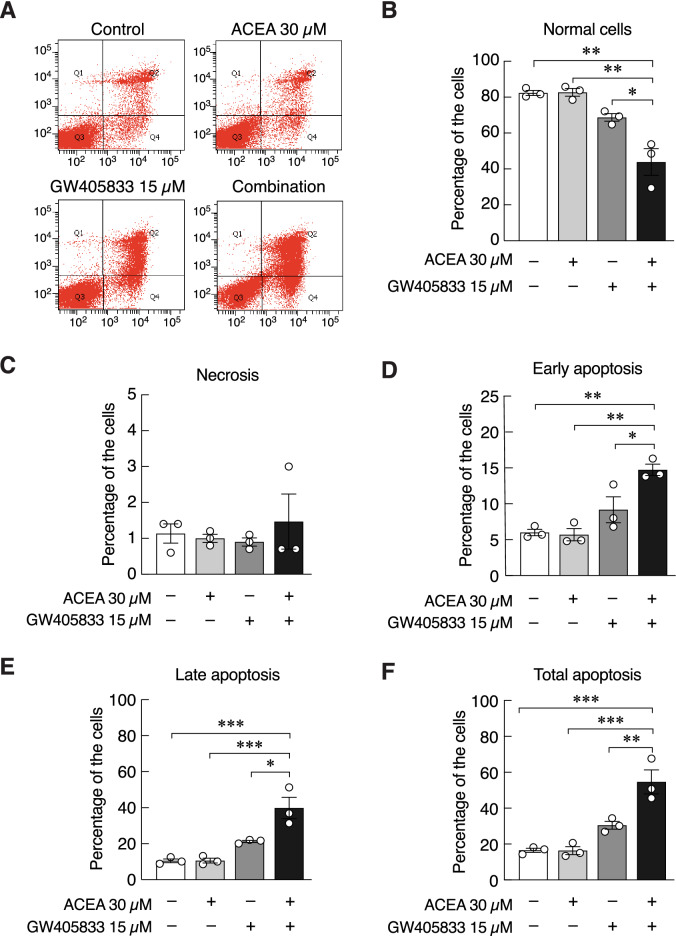
Combining effects of ACEA and GW405833 induced apoptosis in MDA-MB-231 cells (**a**) Apoptotic cell death in cells treated for 48 h with 30 µM ACEA, 15 µM GW405833, or a combination of both agents was analyzed by flow cytometry using Annexin-V-FITC and PI double staining. Quantified data of (**b**) normal cells, (**c**) necrosis, (**d**) early apoptosis, (**e**) late apoptosis and (**f**) total apoptosis, after treatment with CB receptor agonists were shown. Bar graphs represent mean ± SEM of three independent biological experiments. (***p < 0.001, **p < 0.01, *p < 0.05).

To explore whether the enhanced cytotoxicity in MDA-MB-231 cells seen in the combined treatment of ACEA and GW405833 occurred through apoptosis, Annexin-V-FITC/PI double staining assay was carried out. It was shown that the combined treatment significantly increased the proportion of apoptotic cell death in MDA-MB-231 cells (Fig. 5a,b,c,d,e,f). Our results revealed that the percentage of normal cells was significantly decreased in the combined treatment as compared to other groups (Fig. 5b). This corresponded to a significant increase in early apoptotic, late apoptotic and total apoptotic cell proportion in MDA-MB-231 cells (Fig. 5d,e,f). On the other hand, the cells in necrotic phase in all treated groups remained unchanged as compared to the control (Fig. 5c). The finding indicated that the combination of CB1 and CB2 agonists at the ratio of 2:1 led to apoptosis of MDA-MB-231 cells rather than necrosis.

### Combination of ACEA and GW405833 suppressed nuclear factor-κB (NF-κB) signaling pathway in MDA-MB-231 cells

**Figure 6:**
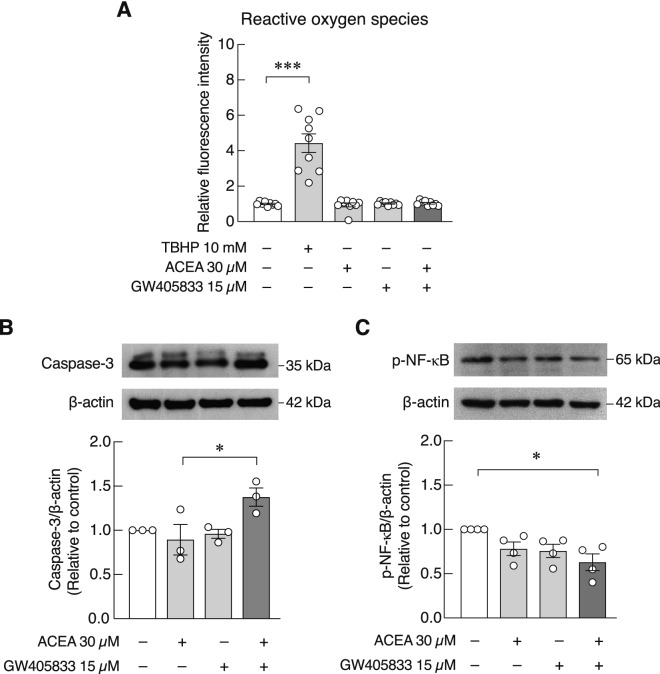
Combination of ACEA and GW405833 did not have effect on ROS but decreased p-NF-κB protein expression and increased Caspase-3 protein expression in MDA-MB-231 cells. (**a**) Cellular ROS in MDA-MB-231 cells treated for 6 h with 30 µM ACEA, 15 µM GW405833, or a combination of both agents was quantified by DCFDA cellular ROS detection assay. As positive control, cells were treated with 10 mM TBHP (tert-butyl hydroperoxide) for 6 h. The expression of (**b**) Caspase-3 and (**c**) p-NF-κB proteins in cells treated for 48 h with ACEA and GW405833 alone or in combination was analyzed by western blot analysis. Columns represent mean ± SEM of three independent biological experiments. (***p < 0.001, *p < 0.05).

Since ROS plays an important role in apoptosis in many cell types^28,29^, we then examined whether cellular ROS level was altered in MDA-MB-231 after exposing to ACEA, GW405833 or their combination. It was shown that cellular ROS levels in all treated cells remained unchanged as compared to control group (Fig. 6a). Furthermore, we examined whether the combined treatment of ACEA and GW405833 affected the apoptosis-associated proteins. Our data showed that the expression of effector caspase-3 was significantly increased in the combined treatment (Fig. 6b and Fig. S1). Because NF-κB is often involved in the regulation of several signaling pathways including apoptosis, we further investigated whether the combined treatment affected the expression of p-NF-κB p65. It was apparent that while the level of p-NF-κB p65 remained unchanged in individual treatments, p-NF-κB p65 was significantly reduced in the combined treatment group by 41.57% (Fig. 6c and Fig. S1).

### ACEA and GW405833 inhibited MDA-MB-231 cell migration

**Figure 7:**
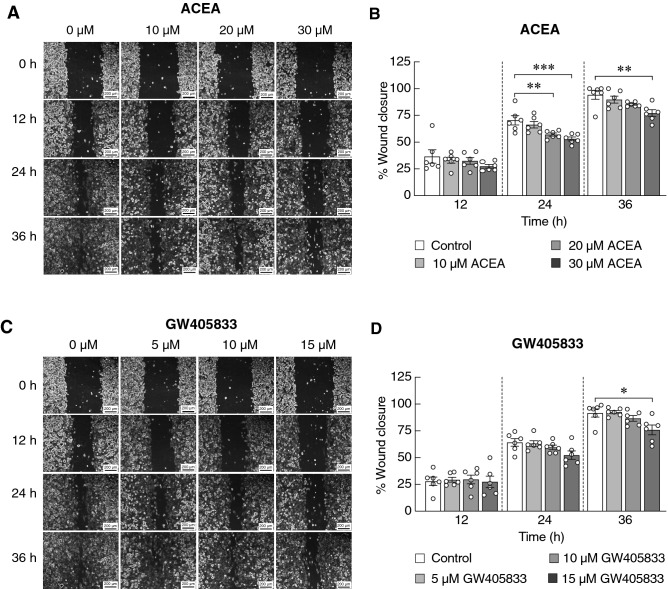
Inhibitory effects of (**a**, **b**) ACEA and (**c**, **d**) GW405833 on MDA-MB-231 cell migration (**a** and **c**) Representative images illustrated MDA-MB-231 cell migration after being treated for 12, 24 and 36 h with the indicated concentrations of the compounds. (**b** and **d**) Percent wound closure representing cell migration was assessed by wound healing assay. Each column represents mean ± SEM of three independent biological replicates with two technical duplicates each. (***p < 0.001, **p < 0.01, *p < 0.05).

Since migration is one of the important characteristics of aggressive cancer and also plays an important role during cancer bone metastasis^30–32^, we further examined whether ACEA and GW405833 could suppress MDA-MB-231 cell migration using wound healing assay. The results showed that the percentage of wound closure, which represented cell migration, was significantly decreased in the presence of 20 µM ACEA at 24 h and 30 µM ACEA at 24 and 36 h (Fig. 7a,b). Similarly, significant cell migration suppression was found in MDA-MB-231 cells treated with 15 µM GW405833 at 36 h (Fig. 7c,d).

**Figure 8:**
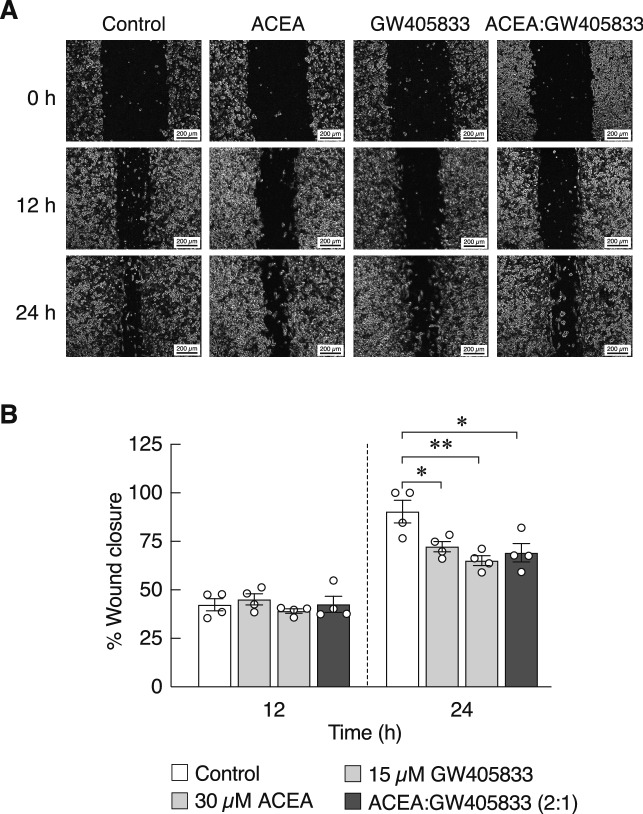
Combining effect of ACEA and GW405833 at ratio of 2:1 on MDA-MB-231 cell migration. (**a**) Representative images illustrated the migration of MDA-MB-231 cells treated with the indicated concentrations of each compound or the combination for 12 and 24 h. Cell migration was assessed by wound healing assay. (**b**) Quantified data is represented as mean ± SEM of two independent biological experiments with two technical duplicates each. (**p < 0.01, *p < 0.05).

As the combined treatment markedly reduced cancer cell viability, we then explored whether the combination of ACEA and GW405833 could further suppress MDA-MB-213 cell migration. This series of experiments confirmed a significant reduction in MDA-MB-231 cell migration by 30 µM ACEA or 15 µM GW405833 given alone at 24 h, whereas a combination of both agonists did not synergistically enhance the inhibitory effects on MDA-MB-231 cell migration (Fig. 8a,b).

## Discussion

ECS has been shown to modulate cancer progression and bone homeostasis. CB expression switch was reported in cancer progression and was probably associated with poor prognosis and cancer aggressiveness^15^. Meanwhile, both CB1 and CB2 were crucial for bone homeostasis^33^ since they could regulate bone formation and resorption. Activation of CB2 promoted osteoblast cell survival and favored bone formation^34–36^ while inhibiting osteoclast differentiation and function—especially in postmenopausal osteoporosis, thus correlating with positive effect of estrogen on CB2 expression in both human and animal model^37^. Mice with CB2 deficiency exhibited a significant reduction in bone mass and develop osteoporosis reflecting crucial roles of CB2 in bone homeostasis. On the other hand, stimulation of CB1 with synthetic agonist resulted in increased osteoclast differentiation and bone resorption activity^36,38^. Nevertheless, CB1 deficiency mice was shown to have increased bone mass only at their early developmental stage (less than 3 months), but they later developed age-related osteoporosis with high level of lipid accumulation in bone. MSCs (bone marrow stromal cells) from these mice had lower osteogenic differentiation but higher adipogenic differentiation ability. Therefore, CB1 was hypothesized to function in MSC differentiation toward osteogenic linage as well^37^. Accordingly, ECS was involved in the regulation of bone remodeling, and the presence of both CB1 and CB2 was important for maintaining bone homeostasis.

Interaction between cancer and bone cells appeared to be crucial during cancer bone metastasis when cancer cells regulated bone cell differentiation and activity to facilitate their colonization in bone. This phenomenon was also evident in breast cancer when breast cancer cells secreted a number of factors, such as transforming growth factor-β_1_ (TGF-β_1_), interleukin 1 (IL-1), IL-6 and IL-11^14,39–41^, all of which suppressed osteoblast differentiation and function. Previous study also reported that CM collected from breast cancer cells induced osteoblast cell death^13^, thus consistent with our finding that breast cancer MDA-MB-231 cell-derived CM inhibited osteoblast-like UMR-106 cell viability. Besides, conditioned media from metastatic breast cancer cell also reportedly induced the expression and secretion of inflammatory cytokines including interleukin (IL)-6, IL-8, monocyte chemoattractant protein (MCP)-1 in osteoblasts. Since these factors were known to attract osteoclast precursor cells and promote osteoclast differentiation, this mechanism could increase bone resorption during breast cancer bone metastasis^42,43^. Interestingly, CB2 agonist (GW405833) pretreatment prevented the toxic effects of MDA-MB-231 cell-derived CM on UMR-106 cells. This result corresponded to the inhibitory effects of CB2 agonists on breast cancer bone colonization and cancer-induced bone loss reported in rodents^44,45^. Our results further showed that CB agonist treatments of breast cancer cells also reduced the activation of NF-κB, which were involved in cancer-bone interaction, presumably by regulating the expression of inflammatory cytokines in breast cancer (as reviewed in Gordon et al.)^46^.

In addition to the positive roles of ECS in bone formation as mentioned previously, our data also suggested another aspect of how ECS could protect bone microenvironment during breast cancer bone metastasis by interfering with cancer and bone interaction, particularly by counteracting the negative effects of cancer cells on osteoblast viability. Other studies also showed that CB2 activation in osteoblasts upregulated the production of RANKL and OPG, cytokines functioning in osteoclast-osteoblast crosstalk^35^. Thus, CB2 agonist in bone microenvironment could facilitate bone formation and compromising the interaction between cancer cells versus osteoclast and/or osteoclasts at the same time. Nevertheless, more studies are required to uncover the mechanism by which ECS prevents the breast cancer cell-mediated osteoblast suppression.

Previous studies reported that the activation of CB1 or CB2 alone suppressed tumor growth and induced cancer cell death^16,17^. Since both CB1 and CB2 ligands were present in bone microenvironment^5,47^, breast cancer cell responses to different combination of CB1 and CB2 agonists were investigated in this study. Both ACEA (CB1 agonist) and GW405833 (CB2 agonist) were shown to suppress the viability of both MDA-MB-231 and UMR-106 cells with MDA-MB-231 cells being more sensitive to ACEA and GW405833 than UMR-106 cells. Interestingly, the growth stimulatory effect of low concentrations of ACEA and GW405833 on osteoblasts was also observed. The expression of CB1 and CB2 on the cell membrane of MDA-MB-231 and UMR-106 cells has previously been demonstrated^38,48^. However, there has been no comparative study on the relative distribution of CB1 and CB2 on the two cell types. With the opposite effects, i.e., growth promoting effect of low concentration of CB agonists on breast cancer and osteoblast cell growth, we hypothesized that the differential responses of MDA-MB-231 and UMR-106 cells to CB agonists was likely to result from different downstream signaling pathways.

Indeed, several cell types in bone microenvironment, e.g., endothelial cells, basophils and macrophages, are able to produce endocannabinoids^49,50^. It is noteworthy that immune response is the very first response when cancer cells metastasize to a new environment including bone^51^. Since cancer cells extravasate from blood vessels to the sinusoidal area of bone, they would be exposed to the endocannabinoid-rich environment in the highly vascularized area of the bone. CB activation has been reported to facilitate immune cell proliferation and activity^50^. Moreover, previous study also showed that the production of endocannabinoids was significantly increased in the activated lymphocytes as compared to the inactivated cells^52^. Therefore, immune activation from cancer metastasis could further upregulate the endocannabinoids production from immune cells at the metastatic site. Taken together, the arriving cancer cells would presumably be exposed to high concentrations of endocannabinoid ligands released from the endothelial and immune cells in the sinusoidal milieu. In other words, only the arriving cancer cells, but not distant osteoblasts, would be compromised by the high concentrations of CB agonists.

Furthermore, potential mechanism underlying the negative effects of CB agonists on breast cancer cell survival was investigated in MDA-MB-231 exposed to each CB agonist alone and the combined treatment. Our findings suggested that simultaneous exposure to both agonists in bone microenvironment might provide stronger protective effects during breast cancer bone metastasis. Our data corresponded to the synergistic effects of CB1 and CB2 agonists on breast cancer growth inhibition and tumor-induced pain suppression reported previously^53,54^. Our results showed that simultaneous activation of CB1 and CB2 enhanced cancer cell death via apoptosis rather than necrosis, and the mechanism involved downregulation of the phosphorylated NF-κB. Apoptotic cell death is often followed by effective cell clearance, which prevents the release of cell content and inflammation within bone environment^55^; therefore, this process should not induce osteoclastogenesis due to proinflammatory cytokine production. Previous studies showed that activation of CB1 and CB2 in breast cancer suppressed ERK1/2 and AKT/mTOR signaling pathways^18–20^. On the other hand, NF-κB, one of the downstream effectors of both pathways, was shown to be associated with cancer cell survival and progression^21–23^. Inhibition of NF-κB activity in breast cancer was also shown to induce apoptosis corresponding to the reduced phosphorylated NF-κB level and enhanced apoptosis in MDA-MB-231 cells treated with CB agonists in this study^56^. Taken together, this study strongly suggested the role of NF-κB pathway in the CB-mediated cancer cell suppression.

In addition, the involvement of ROS production in cancer cell progression and apoptosis was reported^24^. On the other hand, ROS has been known to be associated with several bone diseases by promoting osteoclast activities while suppressing osteoblast function^25^. However, we found that ROS production was not increased in CB agonist-exposed MDA-MB-231 cells in this study. In other words, ECS was capable of inducing breast cancer cell apoptosis in a pathway that did not involve ROS and its harmful effect from ROS on bone homeostasis. Since cell migration was markedly inhibited by CB agonists, it was likely that ECS may also help restrict the spreading of breast cancer cells within bone microenvironment.

In conclusions, we have demonstrated that the ECS—which was present in bone microenvironment—provided a protection against breast cancer bone metastasis and its negative consequence on bone cell survival. Specifically, CB agonists, especially CB2 agonist, was able to prevent breast cancer-induced osteoblast suppression. Each of the two CB agonists or a combination of both could reduce breast cancer cell survival and migration through the NF-κB-dependent pathway. Regarding the limitation of the present study, we realize that a series of studies on the plasma membrane expression levels and binding affinity of CB1 and CB2 receptors might help explain the differential responses of MDA-MB-231 cells vs. osteoblasts to CB agonists. Although additional studies are also needed to confirm the present findings in an *in vivo* model, we have provided evidence to support the novel roles of ECS in breast cancer bone metastasis, thus promoting better understanding of the pathophysiological importance of ECS within bone microenvironment.

## Supplementary Information

Supplementary Information.
