# Supplementary material for: Activation of cannabinoid receptors in breast cancer cells improves osteoblast viability in cancer-bone interaction model while reducing breast cancer cell survival and migration
Source: Sci Rep. 2022 May 5;12:7398. doi: 10.1038/s41598-022-11116-9 (PMC9072415; doi:10.1038/s41598-022-11116-9)
Supplement: Supplementary file 1 — Supplementary Information. [file 41598_2022_11116_MOESM1_ESM.pdf]

Activation of cannabinoid receptors in breast cancer cells improves osteoblast viability in cancer-bone interaction model while reducing breast cancer cell survival and migration

Tueanjai Khunluck[1,2], Kornkamon Lertsuwan[1,3,*], Chartinun Chutoe[3], Supagarn Sooksawanwit[1,4], Ingon Inson[3], Jarinthorn Teerapornpuntakit[1,5], Rutaiwan Tohtong[3], Narattaphol Charoenphandhu[1,4,6,7]

[1] Center of Calcium and Bone Research (COCAB), Faculty of Science, Mahidol University, Bangkok, Thailand
[2] Faculty of Nursing, HRH Princess Chulabhorn College of Medical Science, Chulabhorn Royal Academy, Bangkok, Thailand
[3] Department of Biochemistry, Faculty of Science, Mahidol University, Bangkok, Thailand
[4] Department of Physiology, Faculty of Science, Mahidol University, Bangkok, Thailand
[5] Department of Physiology, Faculty of Medical Science, Naresuan University, Phitsanulok, Thailand
[6] Institute of Molecular Biosciences, Mahidol University, Nakhon Pathom, Thailand
[7] The Academy of Science, The Royal Society of Thailand, Bangkok, Thailand

Type of article: Research article

*** To whom correspondence should be addressed:**
Kornkamon Lertsuwan, Ph.D.
Department of Biochemistry
Faculty of Science, Mahidol University
Rama VI Road, Bangkok 10400
Thailand
E-mail: kornkamon.ler@mahidol.edu

Keywords: apoptosis; bone metastasis; breast cancer cell; cannabinoid receptor (CB); endocannabinoid system (ECS)

Table S1. Fold differences between IC_{50} of each agonist between UMR-106 cells and MDA-MB-231 cells

Compounds	Fold differences between IC_{50} values of UMR-106 / MDA-MB-231			
	**24 h**	**48 h**	**72 h**	**Average**
ACEA	1.54	1.63	1.16	**1.44**
GW405833	6.83	7.18	6.27	**6.76**

Khunluck et al. Activation of cannabinoid receptors in breast cancer cells improves osteoblast viability in cancer-bone interaction model while reducing breast cancer cell survival and migration.

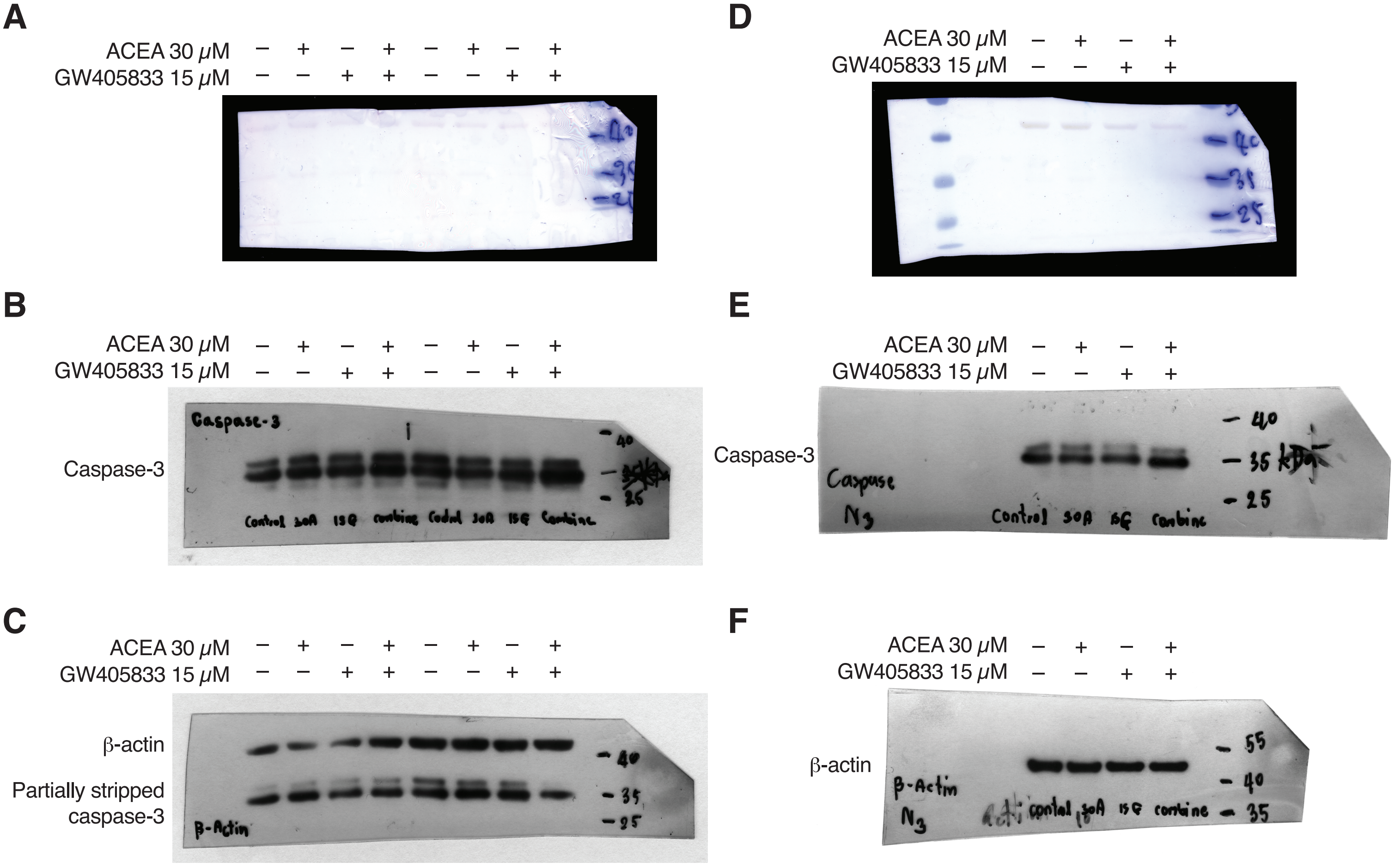

Supplementary Figure S1: Khunluck et al.

Figure S1. Original nitrocellulose membranes and films for western blot images showing the expression levels of caspase-3 and p-NF-κB in MDA-MB-231 cells after treated with 30 μM ACEA, 15 μM GW405833 or combining treatment were illustrated. The labels (e.g., molecular weight) were written before cutting the membranes. As for caspase-3 membrane (A), it was cut by scissors at around 55 kDa. Then, the membrane that contained proteins between ~20 and 55 kDa was hybridized with caspase-3 antibody (MW = 35 kDa). Finally, the membrane was stripped by using stripping buffer (200 mM Glycine, 3.5 mM SDS and 1% v/v Tween-20) before being hybridized with β-actin antibody (MW = 42 kDa) (B–C). Another membrane for caspase-3 was also cut by scissors at 55 kDa (D). Then, the membrane with proteins between ~20 and 55 kDa was hybridized with caspase-3 antibody before being stripped by using stripping buffer. Finally, the same membrane was hybridized with β-actin antibody (E–F). (cont.)

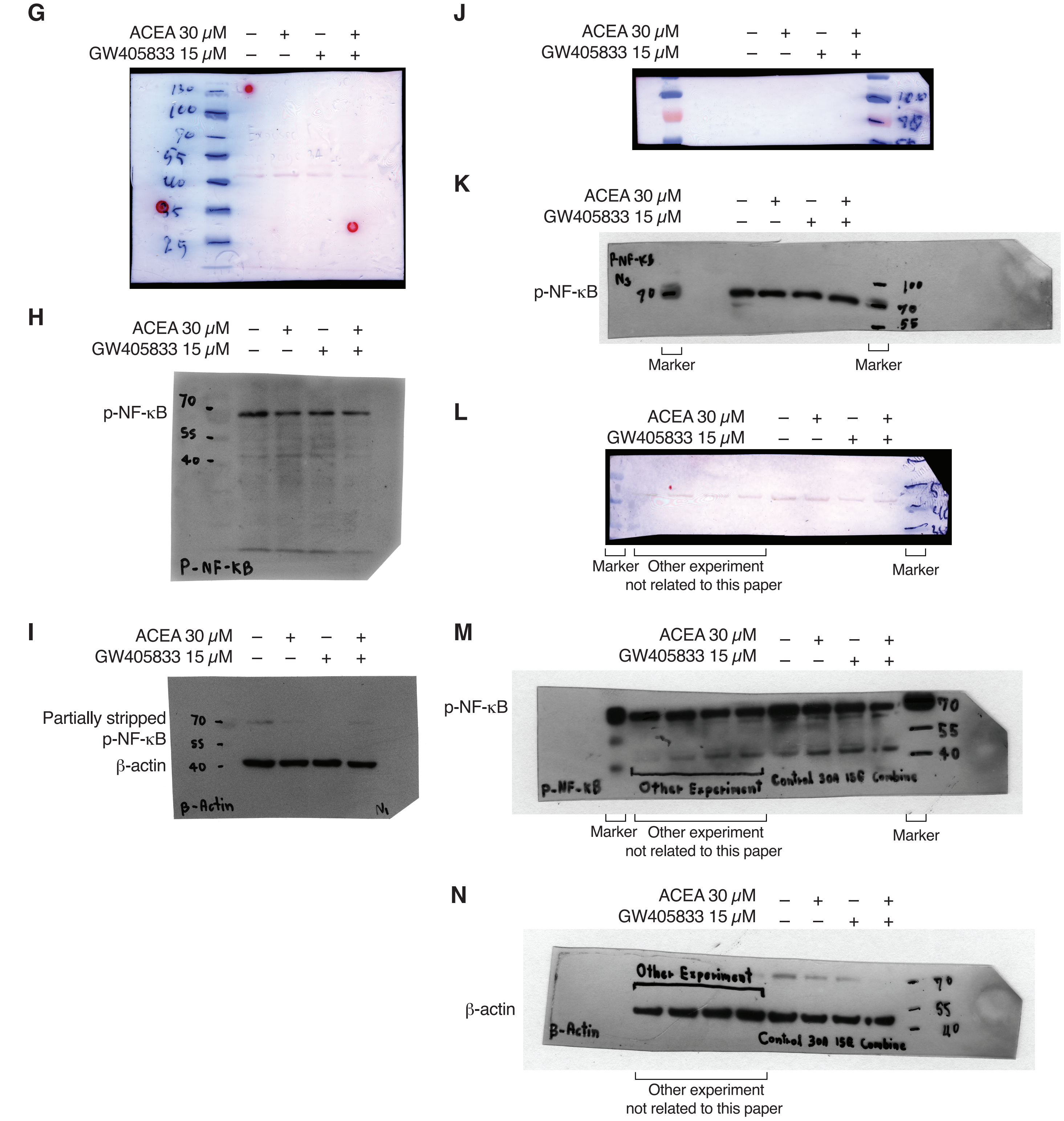

Supplementary Figure S1: Khunluck et al.

Figure S1. (cont.) The membrane was used for phosphorylated NF-κB antibody (MW = 65 kDa) hybridization (G). Then, the membrane was stripped by stripping buffer before re-probing with β-actin antibody (H–I). In (J), the membrane was cut at 55 kDa, and the membrane containing proteins between 55 kDa and ~130 kDa was used for phosphorylated NF-κB antibody hybridization (K). The β-actin blot in (F) was also used as an internal control for this experiment. In (L), the membrane was cut at upper than 70 kDa and lower than 35 kDa. The membrane containing proteins between ~35 kDa and ~70 kDa was used for hybridization with β-actin antibody and then stripped before hybridizing with phosphorylated NF-κB antibody (M–N).
